# 沉默*NGAL*基因对肺癌细胞迁移及侵袭的影响

**DOI:** 10.3779/j.issn.1009-3419.2015.04.03

**Published:** 2015-04-20

**Authors:** 健 唐, 捷 李, 少军 李, 璟波 李, 长海 于, 承泽 尉

**Affiliations:** 1 100071 北京，军事医学科学院附属医院 Afliated Hospital of Academy of Military Medical Sciences, Beijing 100071, China; 2 100048 北京，解放军总医院第一附属医院胸心外科 Department of Cardiothoracic Surgery, the First Afliated Hospital of General Hospital of Chinese People's Liberation Army, Beijing 100048, China; 3 100853 北京，解放军总医院胸外科 Department of Chest Surgery, General Hospital of Chinese People's Liberation Army, Beijing 100853, China

**Keywords:** 肺肿瘤, *NGAL*基因, 迁移, 侵袭, 上皮细胞-间质细胞转化, Lung neoplasms, Neutrophil gelatinase-associated lipocalin, Migration, Invasion, Epithelial-mesenchymal transition

## Abstract

**背景与目的:**

检测NGAL在肺癌组织的表达，观察小干扰RNA（siRNA）沉默NGAL基因表达对肺癌细胞增殖及侵袭能力的影响。

**方法:**

应用免疫组织化学检测肺癌组织中NGAL表达。通过RNAi技术干扰NGAL后，运用定量PCR、Western blot技术，观察干扰NGAL基因效率。干扰NGAL后，MTT检测细胞增殖、Transwell实验检测细胞迁移侵袭能力，细胞划痕愈合能力，免疫荧光及Western blot方法检测上皮细胞-间质细胞转化（epithelial-mesenchymal transition, EMT）相关的蛋白E-cadherin及Vimentin的表达情况。

**结果:**

肺癌中NGAL阳性率高于癌旁组织（*P* < 0.01）。NGAL-siRNA转染组，癌细胞中NGAL基因及蛋白水平表达都受到明显的抑制。NGAL-siRNA转染组细胞增殖能力，迁移及侵袭能力相对于对照组都降低，差异具有统计学意义（*P* < 0.05）。干扰NGAL，E-cadherin的表达升高，Vimentin的表达水平降低。干扰NGAL，MMP-2和MMP-9蛋白表达水平降低。

**结论:**

NGAL在肺癌组织高表达，且在肺癌细胞的增殖、侵袭和迁移及EMT过程中起着重要作用。NGAL可能是肺癌浸润和转移的一个重要参考指标，可为肺癌治疗提供潜在的靶点。

## Abstract (en)

**Background and objective:**

To detect the expression of neutrophil gelatinase-assoeiated lipocalin (NGAL) in the different differentiations of lung cancer tissues and to study the mechanism of invasion of A549 cells affected by NGAL.

**Methods:**

The expression of NGAL was detected by immunochemistry in lung cancer tissue and the tissue around edge of the cancer. The effect of NGAL expression on A549 cells was observed by using qRT-PCR and Western blot. The abilities of invasion and metastasis were evaluated by transwell invasion and migration assay, and cell scratch assay *in vitro*. The protein expression of E-cadherin, Vimentin was measured by immunofluoresence and Western blot.

**Results:**

The positive expression rate of NGAL was 76.32% (58/76) in the lung cancer, 13.3% (4/30) in adjacent tissue by immunochemistry. NGAL expression levels in the lung cancer tissues were significantly higher than that in adjacent tissues. The rate of migration and invasion in NGAL-siRNA group was 60.4%±6.4% compared to 50.5%±4.4% in the control group, there was a significant difference (*P* < 0.05). Vimentin was suppressed, and E-cadherin was upregulated when NGAL was inhibited. MMP-2 and MMP-9 decreased when NGAL was knocked down.

**Conclusion:**

The expression level of NGAL is highly expressed in lung cancer. NGAL may be one of important indicators involved in lung cancer infiltrated and transferred. NGAL might be one of potential targets for lung cancer treatment.

肺癌，又称原发性支气管肺癌，是肺部常见的恶性肿瘤。在全球，肺癌的发病率和死亡率均居癌症之首，而且目前呈现出一种继续上升趋势^[1]^。*NGAL*基因的蛋白编码产物是一种25 kDa的糖蛋白，是1993年由Kjeldsen等^[2]^在中性粒细胞中首先发现，是脂质运载蛋白（Lipocalln）家族的成员之一。肿瘤组织中，如肺癌、结肠癌、肝癌、乳腺癌和胰腺癌等，均能够检测到*NGAL*基因高表达^[3]^。这提示NGAL可能与肿瘤的发生和发展有关^[3-6]^。本研究旨在探讨NGAL在肺癌组织的表达，观察小干扰RNA（siRNA）沉默*NGAL*基因表达对肺癌细胞增殖及侵袭能力的影响。

## 1 材料与方法

### 1.1 一般资料

选择解放军总医院第一附属医院胸外科76例肺癌患者手术切除的肺癌及30例癌旁组织蜡块（2010年5月-2014年4月），所有标本均经多聚甲醛固定，石蜡包埋，5 µm厚连续切片。年龄45岁-72岁，平均年龄62.6岁。

### 1.2 试剂

高转移的肺癌细胞系A549购自中国科学院，培养基RPMI-1640购自Gbico公司，siRNA oligos购自上海吉玛公司，转染试剂X-tremeGENE（Roche公司），鼠一抗NGAL、GAPDH、MMP-2、MMP-9抗体，兔一抗E-cadherin、Vimentin购自Santa Cruz公司；Alex 594/488标记羊抗兔二抗（Jackson公司），HRP标记的二抗羊抗兔、HRP标记的羊抗鼠（北京中杉金桥公司），TRIzol（Invitrogen公司），反转录试剂盒及定量PCR试剂盒（TAKARA公司）；倒置荧光显微镜为Zessis公司、移液器均为Eppendorf公司。免疫组化SP法试剂盒及DAB显色试剂盒均购自北京中杉金桥生物技术有限公司。

### 1.3 方法

#### 1.3.1 细胞培养

A549细胞用RPMI-1640培养液，含10%胎牛血清、100 U/mL青霉素和0.1 mg/mL链霉素的培养液，置37 ℃，5%CO_2_条件下培养。

#### 1.3.2 免疫组织化学

60 ℃烤片20 min后，常规二甲苯脱蜡，梯度酒精脱水；3% H_2_O_2_室温孵育15 min，PBS冲洗3次（每次不少于5 min）；抗原修复后以羊血清工作液封闭，室温孵育30 min加一抗于4 ℃过夜，用与一抗相应的免疫血清IgG代替一抗作为阴性对照；滴加生物素标记二抗，37 ℃孵育30 min，PBS冲洗后滴加辣根过氧化物酶标记的链霉素卵白素工作液；二氨基联苯胺（DAB）显色，自来水充分冲洗后，苏木素复染，常规脱水，透明，干燥，封片。

#### 1.3.3 免疫荧光

石蜡切片脱蜡，水化5 min后，蒸馏水洗3次，3 min/次；微波抗原修复10 min，冷却至室温；滴加正常山羊血清，室温孵育1 h，封闭抗原非特异性结合位点。滴加一抗兔抗Cadherin（1:1, 000），Vimentin（1:1, 000）（Santa Cruz公司）4 ℃孵育过夜。PBS洗3次，5 min/次；滴加Alex594/488标记的羊抗兔IgG（1:1, 000）二抗，室温孵育1 h。PBS洗3次，5 min/次；滴加DAPI，孵育10 min，用来染细胞核；PBS洗3次，3 min/次；抗荧光淬灭封片剂封片。用与一抗相应的免疫血清IgG代替一抗作为阴性对照。用Zessis荧光显微镜拍照。

#### 1.3.4 转染

按Invitrogen公司提供的脂质体Lipofectamine^TM^ 2000转染程序进行。将各组siRNA与转染试剂混合物加入各个培养细胞的六孔板，转染24 h后，更换正常培养液进行实验。NGAL干扰序列为5′-ACATCCGGCAGGACAATGA-3′；对照序列为5′-AACGUACGCGGAAUACUUCGA-3′ ^[7]^。

#### 1.3.5 qRT-PCR检测A549细胞中NGAL mRNA的表达

分别收集对数生长期的转染及对照组A549细胞，按TRIzol试剂盒说明书提取细胞总RNA，用紫外分光光度计分别测定RNA浓度，根据吸光度（optical delnsity, OD）260和OD280的比值，判定其纯度。分别取5 μg细胞总RNA，按反转录试剂盒说明书反转录成cDNA，以cDNA为模板应用。反应条件为：94 ℃预热3 min，然后94 ℃ 30 s，52 ℃ 30 s，72 ℃ 50 s，扩增29个循环，72 ℃延伸10 min。PCR产物进行1%琼脂糖凝胶电泳，通过ImageJ图像分析软件分析各电泳条带的灰度值，以目的条带的灰度值与GAPDH内参照条带的灰度值的比值表示各目的基因mRNA的相对表达量。NGAL的上游引物为5′-GAAGACAAAGACCCGCAAAAG-3′，下游引物为5′-CTGGCAACCTGGAACAAAAG-3′。内参GAPDH的上游引物为5′-ACCACAGTCCATGCCATCAC-3′，下游引物为5′-TCCACCACCCTGTTGCTGTA-3′。

#### 1.3.6 蛋白质印迹检测NGAL蛋白的表达

转染24 h后，A549细胞中加入适量RAPI（PMSF）裂解液，碾碎匀浆，离心提取总蛋白，按照BCA法测定总蛋白浓度，上样量为每孔30 μg总蛋白。按1:4加上样缓冲液后，98 ℃变性10 min。100 V恒压电泳，电泳后进行湿式电转。转膜后用5%牛奶封闭液封闭1 h，一抗鼠来源的NGAL、MMP-2、MMP-9、GAPDH，兔来源的E-cadherin、Vimentin用0.05%TBST按1:1, 000稀释，4 ℃孵育过夜，0.05%TBST洗3次，每次5 min。二抗为HRP标记的羊抗兔IgG，羊抗鼠IgG（1:1, 000），室温孵育1 h，0.05%TBST洗3次，每次5 min。用ECL化学发光法来检测，试验重复3次。

#### 1.3.7 MTT检测A549细胞球的生长抑制率

转染24 h后，取生长良好的A549细胞球消化成细胞悬液，计数并将细胞浓度调整到1×10^3^/孔，接种于96孔板。培养24 h、48 h后，每孔加入10 μL（5 mg/mL）的MTT溶液，孵育4 h后，每孔加入100 μL Formanzan溶液，继续孵育直至显微镜下观察到Formanzan全部溶解。用酶标仪在570 nm处检测各孔OD，空白孔调零。细胞存活率=实验孔/对照孔×100%；细胞生长抑制率=（1-实验组OD值/空白对照组OD值）×100%。

#### 1.3.8 细胞侵袭及迁移实验

用无血清RPMI-1640培养液按1:5稀释Matrigel胶，加人80 μL于Transwell板上室，将Transwell小室置于24孔板中，37 ℃孵育5 h待胶凝固。转染24 h后，将细胞用0.25%胰酶消化，用无血清培养基悬浮并计数。转染NGAL-SiRNA及对照组A549细胞各取200 μL（5×10^5^个/mL）加入上室，加入滤膜直径为8 μm的Transwell小室中。下室加600 µL含有10%FBS的RPMI-1640培养液，细胞继续培养12 h。取出Tanswell上室，PBS洗涤2次，用棉签擦去上表面的细胞，加无水甲醇固定细胞30 min；用0.4%的结晶紫染色液染色2 h。在光学显微镜（×100）下计数穿过基膜的细胞数，每张膜随机选取10个视野，每组设3个重复样品。侵袭率=（实验组侵袭细胞数/对照组侵袭细胞数）×100%。迁移实验只是在Transwell上室不加Matrigel胶，迁移率=（实验组迁移细胞数/对照组迁移细胞数）×100%。

#### 1.3.9 细胞划痕

在24孔板中加入约5×10^5^个细胞，培养24 h后用枪头比着直尺，尽量垂直于背后的横线划痕。用PBS洗细胞3次，加入完全培养基，37 ℃、5%CO_2_条件下再培养24 h后取样，拍照。实验至少重复3次，取平均值。

#### 1.3.10 统计学方法

采用SPSS 11.0统计软件，数据比较采用*t*检验。Western blot条带灰密度用ImageJ软件进行分析，*P* < 0.05为差异有统计学意义。

## 2 结果

### 2.1 免疫组化检测NGAL蛋白在肺癌组织中的表达

通过免疫组化检测NGAL在肺癌中的表达，结果发现NGAL在癌旁组织中表达率为13.3%（4/30），而在肺癌组织中NGAL的阳性信号主要定位于胞质（图 1），其阳性表达率为76.32%（58/76），明显高于癌旁组织中NGAL的表达水平（*P* < 0.01），提示NGAL与肺癌的发生发展有关系。

**1:**
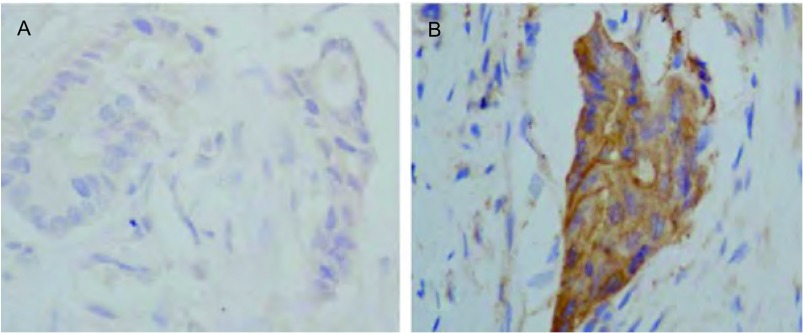
免疫组织化学检测NGAL在癌旁组织（A）及肺癌（B）组织中的表达（×400） The expression of NGAL in adjacent mucosa (A) and lung cancer (B) was detected by immunochemistry (×400)

### 2.2 干扰NGAL的效率

A549细胞转染NGAL-siRNA 24 h后，用qRT-PCR和Western blot检测干扰NGAL的效果，发现control-siRNA转染组NGAL mRNA的表达量为1.05±0.09，NGAL-siRNA转染组NGAL mRNA的表达为的0.35±0.08，明显低于对照组，差异有统计学意义（*P* < 0.01，图 2A）。与NGAL mRNA表达相一致，NGAL-siRNA转染组NGAL的蛋白表达量较对照组的表达量降低（图 2B）。结果提示，NGAL特异性SiRNA从转录和翻译水平高效抑制NGAL表达。

**2:**
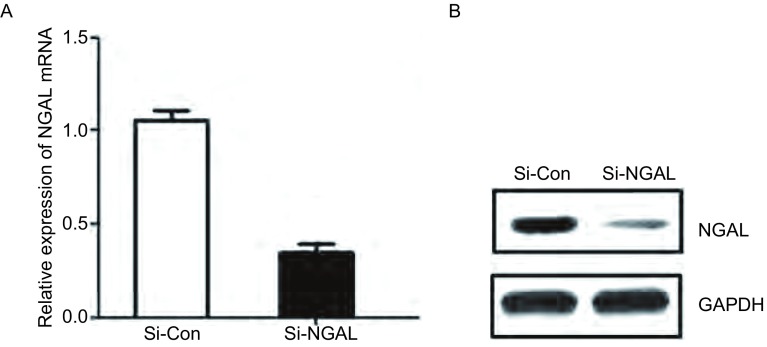
实时荧光定量PCR及Western blot检测干扰NGAL的表达情况。A：qRT-PCR检测表明NGAL mRNA在NGAL-siRNA转染的A549细胞中的表达明显下调；B：Westem blot检测表明NGAL蛋白在NGAL-siRNA转染的A549细胞中表达水平明显下调。 The expression of NGAL mRNA and protein treated with NGAL-SiRNA. A: NGAL mRNA was down-regulated treated with NGAL-SiRNA in A549 cells by qRT-PCR; B: NGAL protein was down-regulated treated with NGAL-SiRNA in A549 cells by Western blot.

### 2.3 干扰NGAL抑制A549细胞的增殖

转染后，分别培养24 h、48 h后，用MTT法检测细胞增殖情况，结果发现沉默NGAL后，A549细胞增殖能力与对照组相比，受到明显的抑制。24 h、48 h细胞存活率为64.92%、56.58%；抑制率分别为35.08%、43.42%，与对照组相比，差异均有统计学意义（*P* < 0.05）（图 3）。

**3:**
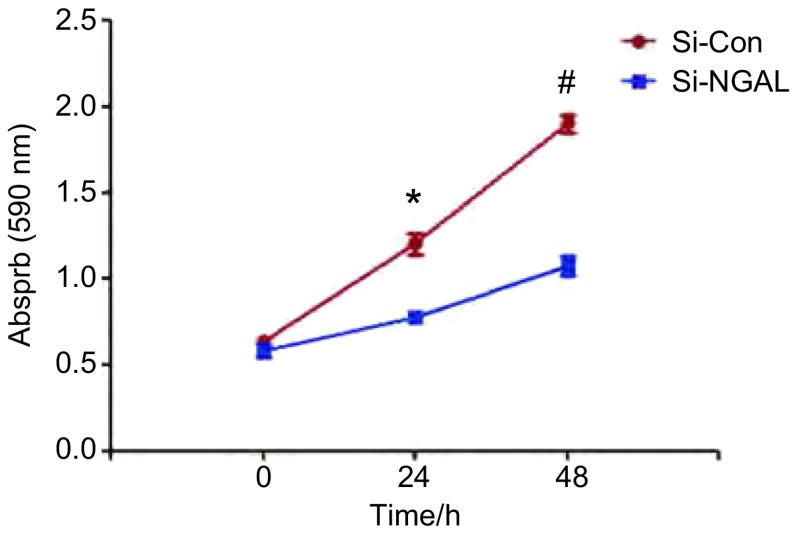
干扰NGAL后细胞在24 h、48 h的增殖情况。与对照组比较，^*^P < 0.05，^#^*P* < 0.01。 NGAL downregulation reduced cell proliferation treated with NGAL-SiRNA after 24 and 48 h. Compared with control group, ^*^P < 0.05, ^#^*P* < 0.01.

### 2.4 干扰NGAL表达抑制A549细胞的体外迁移和侵袭

应用Transwell小室迁移实验发现在接种细胞24 h后，A549细胞穿过8 μm孔径膜的细胞数，对照组为每视野248.0±16.1，而NGAL-SiRNA组穿过膜的细胞数为每视野190.7±14.6（*P* < 0.05）。Transwell小室侵袭实验检测细胞侵袭能力，在接种细胞24 h后，A549细胞穿过Matrigel胶的细胞数，对照组为每视野152.3±11.4，而NGAL-SiRNA实验组穿过Matrigel胶侵袭的细胞数为74.3±11.9（*P* < 0.01）。细胞划痕实验显示，干扰组的细胞迁移较对照组和空质粒组缓慢，划痕后24 h，对照组细胞迁移明显，而干扰组迁移不明显。A549细胞对照组细胞迁移率为81.05%±4.36%，干扰组为41.33%±5.56%，差异有统计学意义（*P* < 0.05）（图 4）。以上结果表明下调NGAL基因的表达后，A549细胞的迁移及侵袭能力明显被抑制。

**4:**
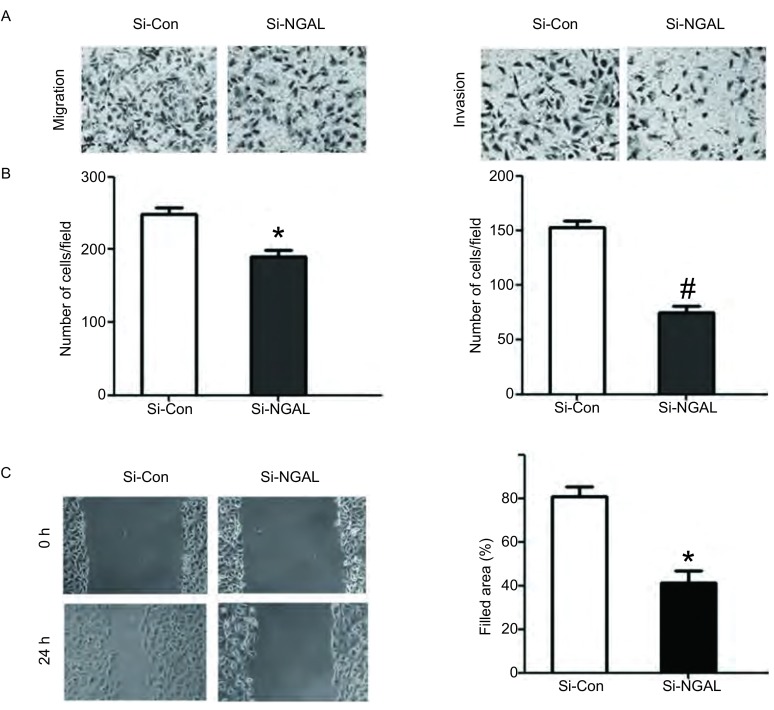
干扰NGAL后，A549细胞迁移侵袭能力的检测。A：干扰NGAL后，细胞迁移能力的比较（*P* < 0.05）；B：干扰NGAL后，细胞侵袭能力的比较；C：干扰NGAL后，对细胞划痕损伤愈合能力的影响。与对照组比较，^*^P < 0.05，^#^*P* < 0.01。 The ability of magration and invasion treated with NGAL-SiRNA or NGAL-Control SiRNA on A549 cells. A: The cell numbers of transwell migration treated with NGAL-SiRNA; B: The ability of invasion treated with NGAL-SiRNA; C: Effect of NGAL-SiRNA transfection on the wound healing ability of A549. Compared with control group, ^*^P < 0.05, ^#^*P* < 0.01.

### 2.5 干扰NGAL抑制上皮细胞-间质细胞转化（epithelial-mesenchymal transition, EMT）相关蛋白E-cadherin和Vimenntin的表达，同时也抑制MMP蛋白的表达

转染后再培养24 h，用免疫荧光及Western blot检测细胞EMT相关蛋白E-cadherin、Vimentin的表达情况。免疫荧光结果显示，干扰NGAL后E-cadherin表达的荧光强度明显强于对照组；而Vimentin的表达强度低于对照组（图 5A）。Western blot检测显示，干扰NGAL后A549细胞中E-cadherin蛋白的表达水平明显于高于对照组，为对照组的2.27倍；Vimentin的表达较对照组明显降低，为对照组的0.23倍，差异均有统计学意义（*P* < 0.05）（图 5）。干扰NGAL后，用Western blot检测了A549细胞中MMP-2和MMP-9的表达，发现确实能够抑制MMP-2和MMP-9蛋白的表达，分别为对照组的0.37倍和0.26倍，差异均有统计学意义（*P* < 0.05）。

**5:**
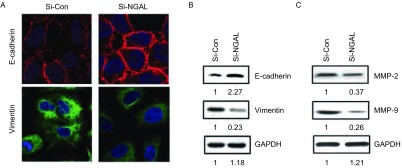
干扰NGAL后，EMT相关蛋白的表达。A：免疫荧光检测干扰NGAL后，E-cadherin和Vimentin的表达；B：Western blot检测干扰NGAL后，E-cadherin和Vimentin的表达；C：Western blot检测干扰NGAL后，MMP-2和MMP-9的表达。 Expression of EMT related marker protein treated with NGAL-SiRNA. A: E-cadherin and vimentin expression was detected by immunoflouresence after treated with NGAL-SiRNA; B: E-cadherin and vimentin expression was detected by Western-blot after treated with NGAL-SiRNA; C: MMP-2 and MMP-9 expression was detected by Western blot after treated with NGAL-SiRNA. EMT: epithelial-mesenchymal transition.

## 3 讨论

肺癌是全球发病率和死亡率最高的恶性肿瘤之一，近年来其发病率在我国呈上升和年轻化趋势，其发生、发展和转移均涉及极其复杂的多基因调控异常过程^[1]^。因此，研究肺癌的病因、发病机制具有重要的临床意义。

NGAL是由Kjeldsen等^[2]^在1993年在人中性粒细胞中发现的。NGAL是Lipocalin家族成员之一。NGAL是分子量为25 kDa的多肽链，含有178个氨基酸残基，定位于人染色体9q34上，全长5, 869 bp。近年来，一些研究提示NGAL在多种肿瘤的发生发展中发挥重要功能，是人类的一种新癌基因。

本研究通过检测NGAL蛋白在肺癌及癌旁组织中的表达发现NGAL在肺癌中阳性表达率远高于癌旁组织，说明NGAL的表达与肺癌发生发展存在一定的关联。已有研究^[3, 4]^发现NGAL高表达肺癌，且于分化程度有关。这与我们报道的结果一致。

有研究发现在肺癌、乳腺癌、卵巢癌和胰腺癌等肿瘤都有NGAL的表达，但没有进一步阐明NGAL在其中究竟发挥着何种作用。为了研究NGAL对肺癌细胞增殖、侵袭迁移能力的影响，本实验通过转染NGAL-SiRNA降低肺癌细胞中NGAL的表达，同时利用MTT、Transwell方法检测抑制NGAL的表达后，肺癌细胞增殖、侵袭迁移能力的变化，结果显示干扰NGAL表达的肺癌细胞增殖能力明显低于对照组；侵袭迁移能力显著降低。我们进一步利用细胞划痕实验发现抑制NGAL的表达后，肺癌细胞的划痕愈合能力明显下调。这些结果都表明NGAL在肺癌细胞的侵袭迁移过程中起着非常重要的作用。许多研究证实在其他癌症中，如乳腺癌、甲状腺癌，干扰NGAL后，能够抑制肿瘤细胞的侵袭迁移，而过表达NGAL后能够增强癌细胞的迁移增殖能力^[5, 6]^。NGAL蛋白的表达水平与肿瘤细胞的S期分数和增殖指数Ki67正相关^[7]^。提示NGAL不仅与肿瘤发生发展有关，还在肿瘤的浸润和转移中具有一定作用，但其作用机制目前尚未明确。

EMT在生物的发育、组织修复和肿瘤转移中发挥着重要作用，是癌症发展和肿瘤转移过程的重要标志性转变EMT的发生是以E-cadherin、细胞角蛋白等上皮性标志物表达下调，Vimentin、N-cadherin等间叶性标志物表达上调为特点，而且发生EMT的肿瘤细胞细胞间迁移和侵袭能力增强^[8]^。本研究发现在抑制NGAL的表达时，Vimentin蛋白表达降低，而E-cadherin蛋白表达明显升高，说明抑制NGAL的表达可抑制A549细胞的EMT转化，进而抑制细胞迁移和侵袭移动。在乳腺癌的研究中同样发现，干扰NGAL能够下调E-钙粘蛋白表达，参与表皮生长因子诱导的EMT，促进肿瘤的浸润和转移^[9]^。而多项研究^[10, 11]^表明NGAL促进癌细胞侵袭行为的另一可能机制是NGAL蛋白能够与MMP-9形成复合物，增加MMP-9活性，促进细胞外基质的降解，促进新生血管的形成，增强肿瘤细胞迁移和侵袭的能力。本研究也证实了敲减NGAL后，MMP-2及MMP-9蛋白表达水平显著降低。还有研究^[12, 13]^表明NGAL蛋白是一种新的铁离子转运载体，可以和铁高亲和力结合，促进肿瘤细胞的增殖。

综上所述，NGAL可作为肺癌发生、进展、迁移和侵袭等过程中一个重要的检测指标及潜在靶点，其在肿瘤发生发展中的机制和意义还有待进一步深入研究。
